# Nanotechnological Antibacterial and Conductive Wound Dressings for Pressure Ulcer Prevention

**DOI:** 10.3390/nano14151309

**Published:** 2024-08-03

**Authors:** Mauro Pollini, Raffaella Striani, Federica Paladini, Aida Kiani, Maria Rosaria Acocella, Carola Esposito Corcione

**Affiliations:** 1 Department of Experimental Medicine, University of Salento, Via Monteroni, 73100 Lecce, Italy; mauro.pollini@unisalento.it; 2 Department of Engineering for Innovation, University of Salento, Via Monteroni, 73100 Lecce, Italy; raffaella.striani@unisalento.it (R.S.); carola.corcione@unisalento.it (C.E.C.); 3 Department of Chemistry and Biology, University of Salerno, Via Giovanni Paolo II, 132, 84084 Fisciano, Salerno, Italy; akiani@unisa.it (A.K.); macocella@unisa.it (M.R.A.)

**Keywords:** nanotechnological coatings, antibacterial properties, thermal conductivity, pressure ulcers, surface treatments, advanced wound dressing

## Abstract

The development of pressure ulcers, associated with increased temperature and moisture in specific areas of the body, and the risk of microbial infections in patients lying in a static position for prolonged periods of time represents a serious issue in medicine. In order to prevent the formation of pressure ulcers, this work aims to present advanced nanostructured coatings developed by three research groups. Nanometric silver, ash and functionalized torrefied biomass were the basis for the treatment of wound dressings to improve thermal conductivity and antimicrobial properties of the conventional cotton gauzes. Each treatment was performed according to its own optimized method. The treated fabrics were characterized in terms of antimicrobial properties, heat transfer, morphology and hydrophobic behavior. The results demonstrated the effectiveness of the deposition treatments also in synergistic actions. In particular, the antibacterial efficacy was improved in all the samples by the addition of silver treatment, and the thermal conductivity was enhanced by around 58% with nanometric ashes. A further step of the study involved the designing of two multilayer systems evaluated using circuit models for determining the total thermal conductivity. In this way, both systems were designed with the aim to guarantee simultaneous efficacy: high antibacterial and hydrophilic properties at the skin level and more hydrophobic and conductive behaviors toward the external environment.

## 1. Introduction

Pressure ulcers (PU) in the elderly population are associated with significant morbidity and mortality and represent a serious economic concern for the health care system [1]. In people aged over 64 years, its prevalence has been estimated at about 0.23%, and from 6.7% to 12.6% among the population receiving home care [2]. Patients lying in a hospital bed are subjected to bodyweight forces that affect soft tissues by compression, tension and shear, thus leading to the onset of pressure ulcers usually localized in the pelvic region [3]. In addition, the skin microclimate, including temperature, humidity and airflow next to the skin surface, has demonstrated an indirect role in the development of PU [3,4,5]. High localized skin temperature, in particular, leads to perspiration and determines skin moisture and wetness, with a negative effect on the soft tissue deformation [4]. When the skin is in contact with a support surface, the body temperature increases due to the accumulation of heat between the support surface and the skin. Combined with increased pressure loading, the risk of pressure ulcer increases due to higher oxygen consumption, carbon dioxide production and metabolic waste products [6]. As a microclimate variable, skin temperature has been shown to increase by 2 °C in 24–96 h before the development of pressure ulcers [5]; moreover, moisture can increase the risk of pressure ulcers by five-fold and can also serve as a source of bacterial contamination [2]. In addition to the superficial contamination of the open pressure ulcers with environmental flora, it is also important to consider that many patients with severe sacral and ischial pressure ulcers are also incontinent of both the bowel and bladder, with a higher risk of additional infections [7].

In this scenario, prevention is the key factor in PU care and requires both a full understanding of the pathophysiology leading to pressure ulcers and strategies to reduce intrinsic and extrinsic risk factors [2]. Any surface in contact with the skin has the potential to alter the microclimate, including textiles [5] and dressing materials. The thermal conductivity of wound dressing materials has a clinical relevance because it expresses the extent of metabolic heat transfer from the tissues under the dressing and through its structure. Lower thermal conductivity is related to higher heat accumulation in the skin under the dressing, which in turn is related to higher risk of PU development [8]. Thus, heat transport plays a fundamental role at the body-medical device interface. The skin temperature is lower than the internal body temperature. When lying down, the sacral region is usually the area most prone to the formation of bedsores, as the skin temperature rises by about 4 °C in a few hours due to the accumulation of heat, not only from the contact of the skin with the mattress, but also from any bandaging [9]. Promoting thermal conductivity, in fact, is an important physiological benefit in terms of wound prevention and treatment [10]. Heat transfer control is a particular topic that involves several research studies in the medical field due to the importance of avoiding overheating in the correspondence of wounds and injury. Bhargava et al. demonstrated that a computational thermal model can explain thermographic findings related to pressure ulcers [11]. Temperature distribution in the skin is dominated by thermal conductivity. With this purpose, the influence of thermal conductivity in temperature regulation during wound healing was investigated by Jain et al., who elaborated a mathematical model for depicting the variation in tissue temperature during the localized wound healing process [12]. The combination of heat and microclimate conditions inspired the studies by Uzun et al. who designed and developed novel “all-in-one” collagen-booster therapeutic nonwoven wound dressings, carboxymethyl cellulose/polylactic acid-based, confirming that the thermal conductivity of fabrics in the wet state was significantly higher than in the dry state, due to the thermal conductibility of the water [13]. In this regard, it is necessary to consider that there are also open-cell polyurethane foam dressings that have very low thermal conductivity (about 0.04 W/mK in dry conditions) due to the air trapped in the micropores of the foam. The dressing becomes wet when it comes into contact with the wound. As water has a thermal conductivity of 0.6 W/mK, sweat or wound exudate will increase the thermal conductivity of the wet dressing. Therefore, the dressing is more effective at transferring heat away from the skin when it is moist [14,15].

Nevertheless, it is very important to not neglect that the moist and warm wound environment also provides favourable conditions for microbial growth, which may initiate an infection if unmanaged [16]. Bacterial infection is the most common complication associated with pressure ulcers and may result in cellulitis, abscess formation, bursitis and osteomyelitis of the bone underlying the wound bed [17]. Thus, the prevention of bacterial contamination in PU management is essential. Several studies report interesting results about the antibacterial properties of wound dressing, developed by means of different technologies. One example is represented by double cross-linked hydrogel wound dressing 3D bioprinted to form living skin tissue with high precision tailoreable for different anti-bacterial wound dressing applications [18]. An other one is the poly(lactic-co-glycolicacid)/andrographolide-mesoporous silica nanoparticles nanofibrous membrane wound dressing produced by electrospinning that shows an efficient wound-healing ability due to epidermal cell adhesion and reduced inflammation process [19]. Several approaches used for producing conductive wound dressings adopt specific methodologies that require the employment of complex materials and processes [8,20,21].

The novelty of this work consists in the development of an advanced wound dressing for prevention of PU growth by acting on both heat accumulation and bacterial contamination by employing simple and easily scalable process methods. At this purpose, three different technologies developed by three research groups have been adopted to deposit functional coatings onto conventional cotton gauzes for improved thermal conductivity and for antibacterial properties.

In particular, in order to achieve higher thermal conductivity than that of the cotton substrate, coatings based on nanometric silver, nanometric ashes and nanometric functionalized torrefied biomass have been deposited; in order to achieve antibacterial properties, a durable nano-silver deposition technology has been adopted on the plain cotton dressing and on the substrates treated with the other technologies [22]. Then, the chemical-physical properties of the different substrates have been evaluated and the antibacterial effectiveness has been tested, both singularly and in synergistic action.

## 2. Materials and Methods

### 2.1. Preparation of the Samples

Conventional cotton gauzes (100% cotton, sterile medical device, size 10 × 10 cm) were used as the substrate for testing the performances of the materials derived from three different technologies developed by three research groups in terms of antibacterial capability, thermal conductivity and water repellence. The coating realized using the three different technologies provided the deposition of the materials on the gauzes by physical sorption and by photo-assisted chemical-physical reaction.

#### 2.1.1. Silane-Ashes Coating Deposition Technology

The treatment of the cotton gauzes was carried out by impregnation with a silane–ashes-based mixture. A sol-gel reaction, in acid conditions (pH 1–3), between the alkoxide precursor tetraethoxysilane (TEOS) and the 3-(trimethoxysilyl)propyl methacrylate, both supplied by Merck, was carried out in order to develop the silane matrix in which a content equal to 3 wt% of dry nanometric ashes was dispersed by stirring at room temperature for 30 min. The ashes, employed as filler, were derived from the biomass pyro-gasification plant CMD ECO20, developed by Costruzioni Motori Diesel S.p.A. (CMD). The filler in nanometric scale was obtained according to a green procedure developed by some of the authors and reported in previous works [23,24].

Each cotton gauze was immersed in the formulation for 10 min and then dried at room temperature for 48 h. The treated cotton specimens were then washed in deionized water and dried at room temperature for 24 h. The samples treated according to this method were labelled as “SAt”.

#### 2.1.2. Functionalized Torrefied Biomass Deposition Technology

The treatment of the cotton gauzes was carried out by impregnation with a functionalized torrefied biomass (FTB) aqueous solution. The FTB was prepared according to a green procedure previously reported [25] based on the mechanochemical functionalization of torrefied biomass with dodecylphosphonium bromide as an antimicrobial agent. Specifically, torrefied biochar and dodecylphosphonium bromide were mixed in a silicon nitride jar with a ratio of 1:2 followed by grinding for 2 h. The product was provided in 92% mass yield. The torrefied biomass derived from Populus nigra was produced in a fixed-bed reactor at a constant flow of nitrogen (N2 = 12 slm) under slow heating, with conditions (10 °C min^−1^) up to the final temperature of 285 °C. The dodecylphosphonium bromide was supplied by Merck and used without any further purification. The cotton gauzes were cut (3 layers) into square samples (4 cm × 4 cm) and immersed in FTB aqueous solutions (2 wt% and 4 wt% ) under stirring. After 3 h, they were taken from the solutions and washed with distilled water to remove excess FTB from the surface. The obtained samples were allowed to dry at room temperature for 24 h. The samples treated according to this method, with aqueous solutions of FTB equal to 2 wt% and 4 wt%, were labelled as “FTB1t” and “FTB2t”, respectively.

#### 2.1.3. Silver Deposition Technology

**Figure 1:**
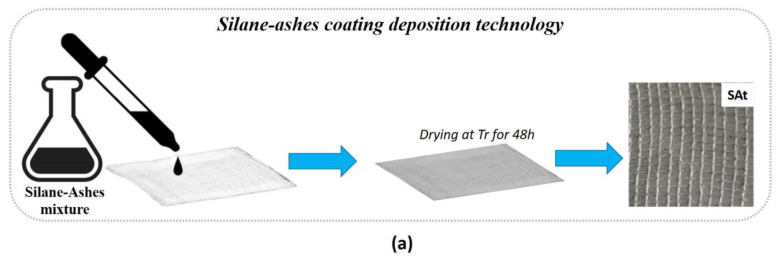

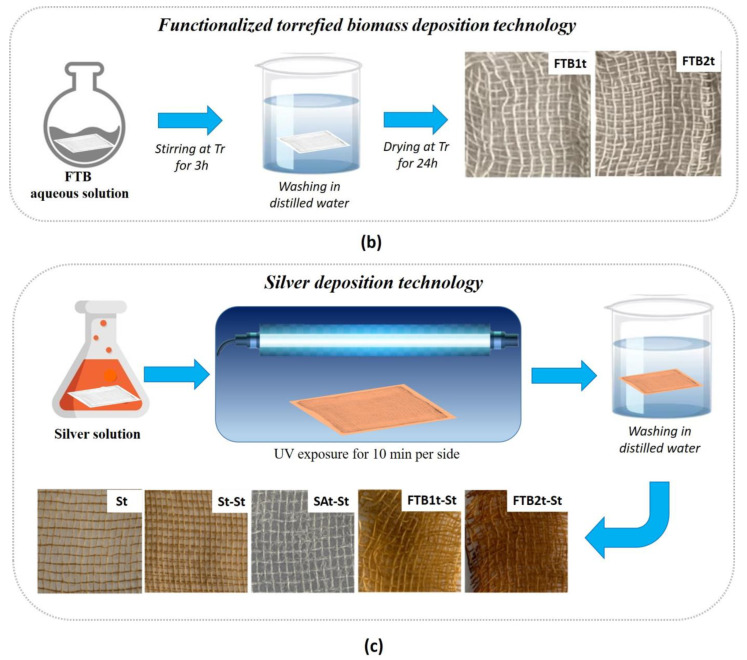
Schematic representation of the silane-ashes coating (**a**), functionalized torrefied biomass (**b**), and silver (**c**) deposition technologies and relative images of the treated samples.

A silver deposition technology was used to confer antibacterial capability to the wound dressing. The process is based on the photochemical deposition of silver nanoparticles [22] and consists of three stages, namely, (i) the preparation of the impregnating silver solution; (ii) the deposition of the silver solution onto the substrate through dip coating or spray coating; and (iii) the exposure of the wet substrate to ultraviolet (UV) radiation (emission peak at λ= 365 nm, power 500 W/m^2^), which allows the conversion in situ from a silver precursor to metal silver nanoparticles. Each stage requires the proper definition of the related process parameters. At the first stage, the composition of the silver solution has to be defined by selecting the percentage of the silver precursor (silver nitrate), the photo-reducing agent (methanol) and the solvent (deionized water). In this work, a silver solution comprising 1 wt/wt% silver nitrate (Sigma Aldrich (St. Louis, MO, USA), ACS reagent, ≥99.0%), 10 wt/wt% methanol (Sigma Aldrich ACS reagent, ≥99.8%) and 89.9 wt/wt% deionized water was prepared. Then, the cotton gauzes were impregnated through dip-coating (stage ii) and finally exposed to the UV lamp (Jelosil) for 10 min per side at a distance of 20 cm (stage iii) [26]. The silver-treated substrates were washed with deionized water to avoid the potential presence of non-reacted silver precursor and labelled as “St” for characterization. A second treatment was also performed on these samples by using the same process parameters (samples were labelled as “St-St”) on the substrates treated through the other technologies in order to evaluate a potential combined effect on the properties of the material (samples were labelled as SAt-St and FTBt-St, where the letters “St” refer to the additional silver treatment). Figure 1 collects the pictures of all the samples prepared through a single treatment (samples “St”, “SAt” and “FTBt”) and through a second silver-based treatment (samples “St-St”, “Sat-St” and “FTB2t-St”).

### 2.2. Characterization Techniques

#### 2.2.1. Characterization of the Fillers

The morphology of the ashes filler employed for developing the silane-ashes based coating was investigated in terms of microscopic and granulometric analysis by using, respectively, the scanning electron microscopy SEM (Evo40-Zeiss) and both the multi-angle laser scattering (MALS) (CILAS1190 particle size analyzer) and dynamic light scattering (DLS, Zetasizer-Malvern). The ashes were also studied by means of energy-dispersive X-ray (EDX-Bruker, XFlash detector 5010) and thermo-gravimetric analysis (TGA-TA Instruments), both performed to evaluate the composition.

The nanometric size of the silver particles and functionalized torrified biomass deposited onto cellulosic fibers has already been demonstrated by SEM in previous works, where also the strong adhesion of the coating to the substrate and the homogeneous distribution of the particles were assessed [27].

The nanometric size and the ability to be uniformly dispersed on the cotton fabric of functionalized torrefied biomass has already been confirmed by SEM images in a previous paper [25]. The functionalized torrefied biomass (FTB) was also characterized by FTIR spectra, collected using a Bruker Vertex70 FTIR spectrometer, which was equipped with a deuterated triglycine sulfate (DTGS) detector and a KBr beam splitter. KBr pellets were utilized during the measurements. To ensure accurate frequency readings, the frequency scale was internally calibrated to 0.01 cm^−1^ using a He-Ne laser. In order to minimize noise, signal-averaging was employed by performing 32 scans and subsequently reducing the overall noise level. Wide-angle X-ray diffraction (WAXD) was obtained through an automatic Bruker D2 phaser diffractometer, in reflection, at 35 kV and 40 mA, using nickel-filtered Cu Kα radiation (1.5418 Å), was also employed.

#### 2.2.2. Characterization of the Gauze Specimens

**Figure 2:**
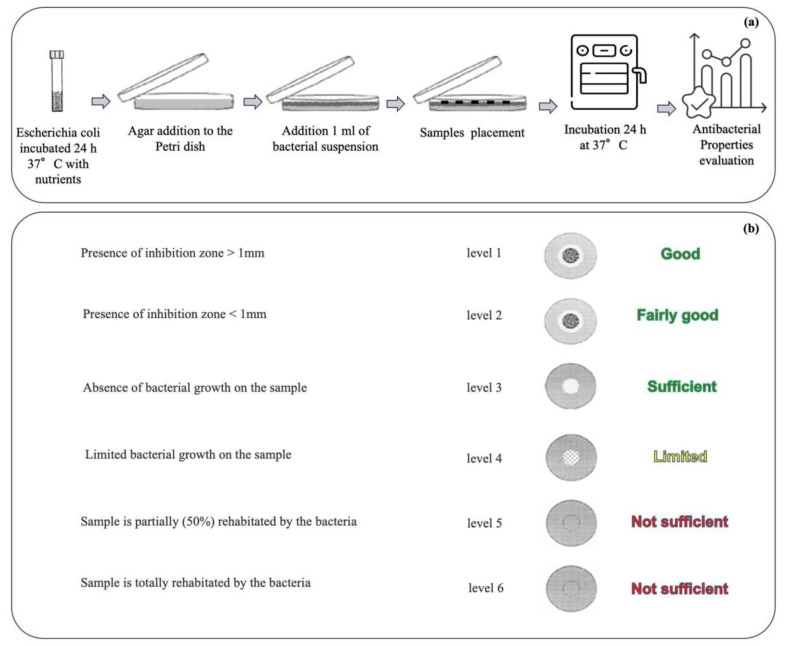
Agar diffusion test experimental protocol according to the Standard SNV 195920-1992” (**a**) and levels of antibacterial capability provided by the Standard (**b**).

The antibacterial capabilities of the materials treated by using the different technologies, alone and in combination, have been characterized from a microbiology point of view through agar diffusion tests, performed according to the Standard SNV 195920-1992 (Figure 2a). Briefly, the samples (size ~3 cm^2^) were incubated in contact with bacteria (Escherichia coli, ATCC 25922, inoculating bacterial density 2 × 108 CFU/mL) on agar plates at 37 °C overnight, and, after incubation, the size of the inhibition area to bacterial growth was evaluated in comparison with the untreated sample according to the levels of antibacterial efficacy reported in the Standard (Figure 2b). In particular, an inhibition zone larger than 1 mm indicates a good level of antibacterial efficacy, while a sample fully covered by bacteria is associated with an insufficient level. Intermediate levels are also provided by the Standard.

Hydrophobicity measurements were performed on untreated and treated cotton gauzes by using bidistilled water (surface tension ɣ= 72.1 mN m^−1^) using the First Ten Angstroms FTA1000 Quick Start. The analyses were performed at room temperature by means of the sessile drop technique. At least five measurements of each specimen were performed, averaging the results to estimate the standard deviation.

Thermal conductivity (TC) was estimated by Differential Scanning Calorimetry (DSC) by means of a Mettler Toledo TGA/DSC1 StareSystem. Indium (Tm = 156.6 °C) was chosen as a sensor material by placing it onto the fabrics. A heating ramp was run at 10 °C min-1 up to the melting of Indium. Three measurements for each sample were performed, averaging the results to estimate standard deviation. The Flynn and Levin’s method [28] was employed for calculating the TC from the thermal resistance of the fabric samples (*R_s_*), which is defined according to Equation (1):

(1) $$R_{s}=R^{’}-R$$

where *R′* and *R* are the thermal resistance determined by the slope of the Indium melting peak placed on top of the fabric sample and without the fabric, respectively. *TC* was estimated according to Equation (2):

(2) $$TC=\frac{L}{A(R^{’}-R)}=\frac{L}{AR_{s}}$$

where *L* is the sample thickness and *A* is the contact area between the sample and the Indium sensor.

**Figure 3:**
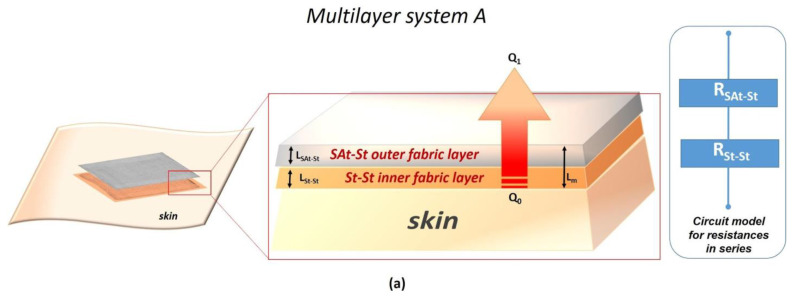

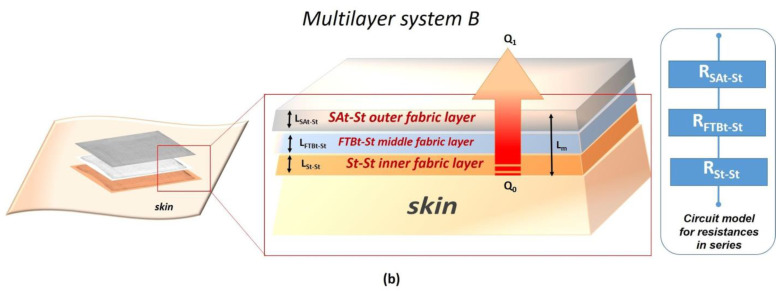
Schematic representation of multilayer A (**a**) and multilayer B (**b**) and the relative circuit model for resistances in series.

In order to predict the thermal conductivity of two multilayer systems based on the combination of the developed technologies, the circuit model for resistances in series was considered [29]. The mathematical model proposed in this work has been simplified by considering both convective and radiative heat transfer as irrelevant contributions, the heat transfer in a textile fabric being mainly dependent on thermal conduction and in a minor part (20%) from radiation [30]. Moreover, the model was designed for still air conditions with adherent layers of the device. Hence, only conductive heat transfer was calculated for predicting the conductive properties of the multilayer systems. In detail, the heat flow was considered only in one direction from higher temperature (lesioned skin) to lower temperature (environment) perpendicularly to the fibers of cotton gauze, as schematized in Figure 3a for multilayer A and Figure 3b for multilayer B with the relative circuit model for the resistances in series.

According to the circuit model for the resistances in series, the total thermal resistance for conduction for each multilayer system can be expressed as follows:

(3) $$R_{m}=\sum_{l=1}^{n}R_{l}$$

where *R_m_* is the total thermal resistance of the multilayer; *R_l_* is the thermal resistance of the single layer; and *l* is the number of layers, where *l* = 2 for multilayer system *A* and *l* = 3 for multilayer system *B*.

Considering the total thermal resistance of the multilayer *R_m_* as the sum of the thermal resistance of each layer, the total thermal conductivity due to heat conduction of the multilayer systems can be determined according to Equation (4) for multilayer system A and Equation (5) for multilayer system B:

(4) $$\frac{L_{mA}}{k_{mA}}=\frac{L_{SAt-St}}{k_{SAt-St}}+\frac{L_{St-St}}{k_{St-St}}$$

(5) $$\frac{L_{mB}}{k_{mB}}=\frac{L_{SAt-St}}{k_{SAt-St}}+\frac{L_{FTBt-St}}{k_{FTBt-St}}+\frac{L_{St-St}}{k_{St-St}}$$

where *L_mA_* and *L_mB_* are, respectively, the total thickness of the multilayer system *A* and multilayer system *B*; *k_mA_* and *k_mB_* are, respectively, the total thermal conductivity due to conduction of the multilayer system *A* and multilayer system *B.*

## 3. Results and Discussion

**Figure 4:**
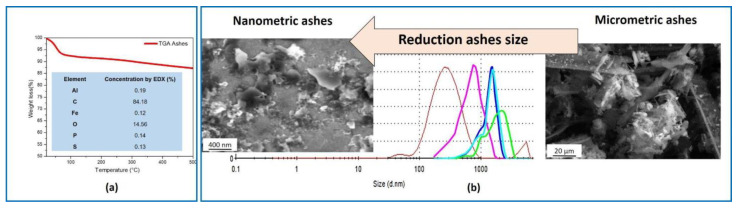
The TGA and EDX element’s concentration of ashes (**a**); SEM images and granulometric curves from micrometric to nanometric ashes (**b**).

From the EDX analysis (inset in Figure 4a) the most abundant element in the ashes was the carbon (>84 wt% ), followed by the oxygen (around 14 wt% ). Impurities of negligible concentration (<0.2 wt% ) such as, aluminum, iron, phosphorus and sulphur were also detected. The TGA (Figure 4a) evidenced an initial loss of absorbed water within 100 °C and a second step at around 250 °C, probably related to the degradation of oxygenated functional groups. In order to obtain nanometric ashes for developing the silane–ashes-based coating, a method that combines ball milling and sonication techniques, previously developed in [23], was applied. As shown by the SEM images and the granulometric curves reported in Figure 4b, the procedure was efficient to reduce the size of the ash particles from the micrometric to the nanometric scale. In fact, a mean diameter of (8.87 ± 1.01) µm was recorded at the start of the process, reaching a mean diameter of (302 ± 149) nm at the end.

**Figure 5:**
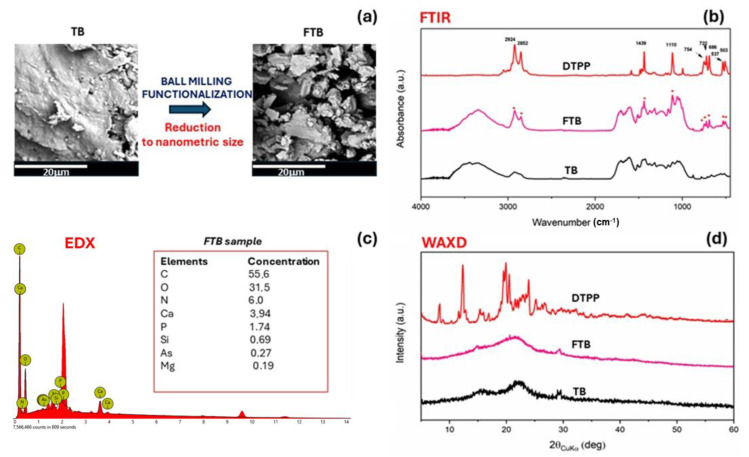
SEM image (**a**) and EDX (**c**) of functionalized torrefied biomass (FTB), FTIR (**b**) and WAXD (**d**) of torrefied biomass (TB) and functionalized torrefied biomass (FTB).

The FTB adduct was synthesized by a mechanochemical approach, providing an ionic functionalized torrified biomass (FTB) with DTPP (dodecyl phosphonium bromide). As confirmed by FTIR, the phosphonium salt was incorporated in the carbon structure with an uptake of 13 wt%, as a result of the EDX analysis. The absence of the DTPP crystalline peaks on the carbon surface rules out the possibility of residual phosphonium salt on the surface (Figure 5). The reduction from millimeter-to-nanometer size through ball milling was evidenced by SEM images, as reported in Figure 5.

**Figure 6:**
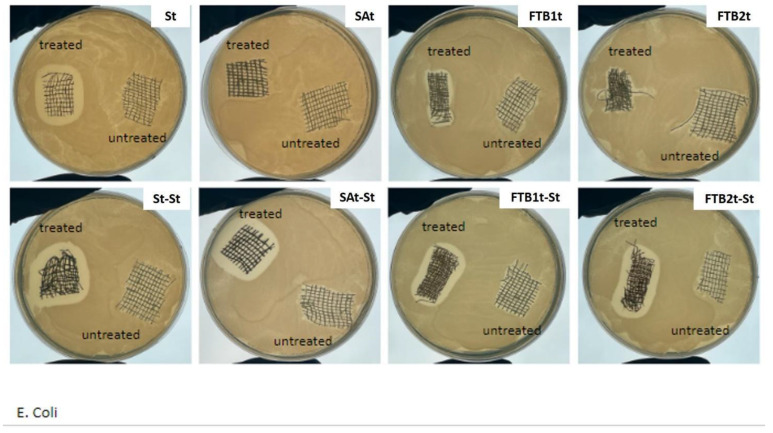
Microbiology tests on the samples treated with the different technologies, alone and in combination with the silver deposition treatment, in comparison with the untreated cotton gauze.

The results of microbiology tests reported in Figure 6 clearly indicate different levels of antibacterial capabilities associated with the different technologies adopted for the treatment of the textile substrates. The silver treated samples St and St-St demonstrated good antibacterial activity even after one single treatment, as visible in Figure 6 where an inhibition area larger than 1 mm can be observed in comparison with the untreated gauze. Moreover, the size of this area appears similar between samples St and St-St, indicating that a single treatment can be preferred in terms of the cost/effectiveness ratio. On the other hand, while the sample SAt did not demonstrate any antibacterial efficacy, it became antibacterial after the silver treatment, thus demonstrating the compatibility between the technologies and also additional antibacterial properties deriving from the presence of silver. An intermediate situation can be observed in relation to samples FTBt. Before the addition of silver, the FTBt samples exhibited an antibacterial activity lower than that of the St samples, which increased after the second treatment with silver, thus achieving a level of antibacterial efficacy comparable with St and St-St. It has to be noted that for both 2wt% and 4 wt% FTB loading (i.e., FTB1t and FTB2t, respectively), the total amount of antibacterial molecules corresponds to 13wt% of the FTB filler. Therefore, under these conditions, the presence of even a lower antimicrobial activity is relevant. The silver deposition technology adopted in this work allows the achievement of antimicrobial capability even with very low amounts of metal silver nanoparticles deposited onto the textile substrate. In a previous work where two different percentages of silver precursor were tested, three characterization techniques were tested to define the content of silver adhered to the cotton dressing. In particular, thermogravimetric analyses (TGA), energy dispersive X-ray spectroscopy (EDX) and UV–Vis–Near IR spectrophotometric analysis with integrating sphere were performed on treated and untreated samples. All results confirmed an amount of silver consistent with the percentage of silver precursor in the silver solution, and the reflection spectra also confirmed the nanosized dimension of the silver particles [22].

**Figure 7:**
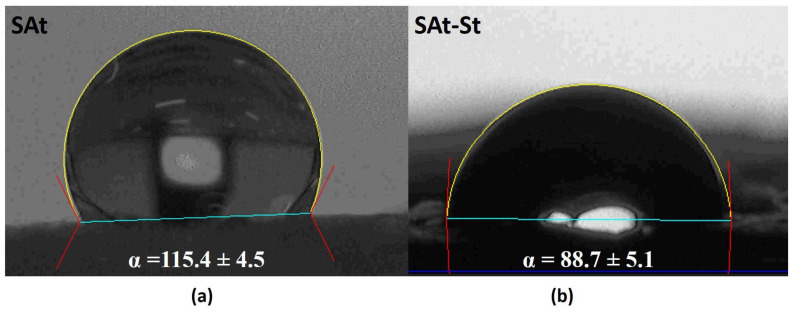
Static contact angle of SAt (**a**) and SAt-St (**b**) gauzes.

In order to evaluate the hydrophilic/hydrophobic properties of the fabrics treated by the three technologies, static contact angle measurements were performed. As expected, as reported in Table 1 and shown in Figure 7a,b, only the samples treated with silane–ashes-based coatings showed a hydrophobic behavior compared to the others, which demonstrated an instantaneous absorption of water drops. Nevertheless, even if the addition of the silver coating on the silane–ashes-based treatment increased the thermal conductivity, the wettability of the surface also changed by decreasing the contact angle values by about 22%.

**Figure 8:**
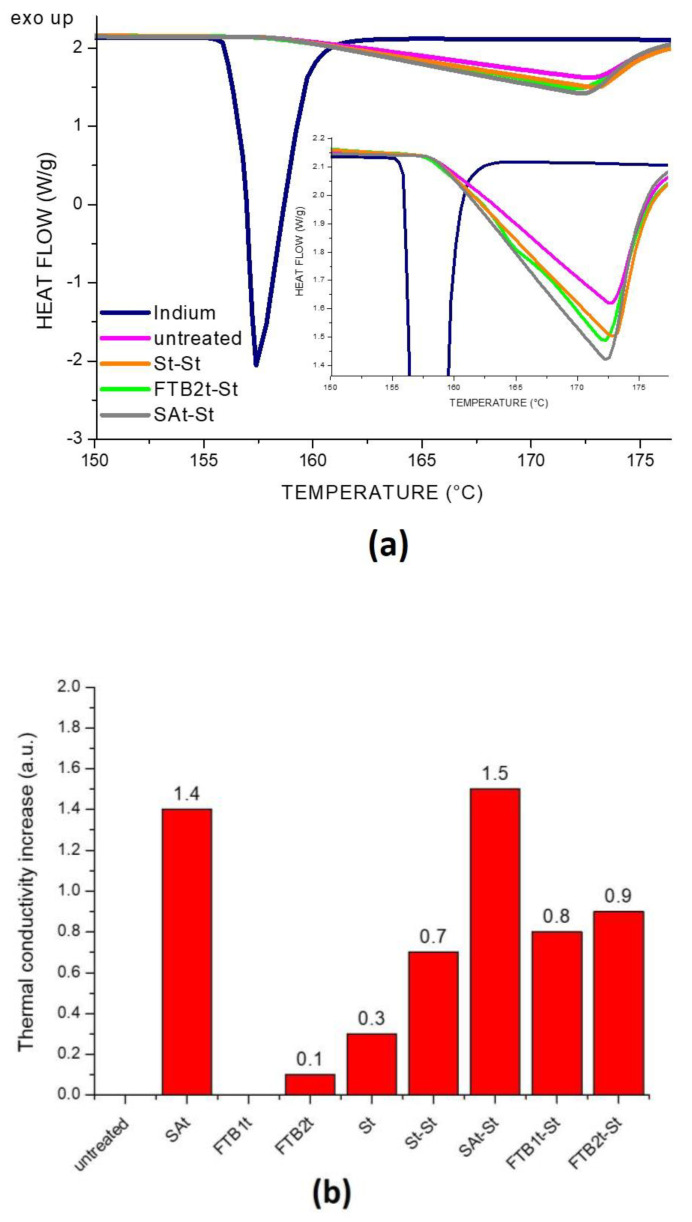
DSC thermograms (**a**) and thermal conductivity evaluation (**b**) of untreated and treated fabrics.

Thermal conductivity analyses, evaluated by DSC, demonstrated an improvement in conductive properties for all the technologies with respect to the untreated cotton gauze. By considering the values reported in Table 1 and the thermograms of the most conductive specimens in Figure 8a, it is clear that the type of treatment modifies the capability of the fabric to be more or less conductive. In particular, as shown in Figure 8b and by the values in Table 1, the treatment with silane–ashes-based coating is the most efficient in terms of thermal conductivity, followed by the silver-based deposition that improved the conductive properties of all the gauzes. It should be highlighted that, despite the high thermal conductivity capabilities of the silver (i.e., 420 W/mK), in this study, the amount deposited on each gauze was very low. Enhanced conductivity performances were also obtained by combining different treatments, such as silane–ashes and silver-based deposition. In fact, the highest values are related to SAt and SAt-St, demonstrating the capability of the silane–ashes-based coating to be conductive thanks to the presence of the carbonious filler (i.e., 3 wt% ). FTBt samples, instead, despite the presence of a carbon content in the solution, showed an effect in terms of conductivity only in co-presence with silver. This could be explained by the torrefaction process of biomass that allows the preservation of many oxygen functional groups on the surface and reduces the percentage of aromatization, thus affecting the conductivity.

Considering that the three approaches developed are easily scalable and do not require toxic materials, it can be stated that the enhancement of thermal conductivity values in a range of 21–60%, for single or double coatings, follows the behavior of specific hydrogel wound dressings [21], as well as tissues treated with solvent-based polymeric solutions [31], even though the latter are enriched with other kinds of nanofillers, requiring complex technologies for the synthesis and application.

The total thermal conductivity of both the multilayer systems calculated according to Equations (4) and (5) and the circuit model for in series resistances is reported in Table 2.

Along with antibacterial properties and thermal conductivity, the different hydrophilic/hydrophobic behaviour of the developed coatings are of interest for the development of the multilayer device (Figure 3a,b).

A wide range of dressings has been studied for the prevention of pressure ulcers, including films, hydrocolloids and foams as the most commonly reviewed dressings. Emollients and film-forming barriers have also been used as protection of skin from friction, shear and damages [32], while good efficacy has also been demonstrated by silver ion dressings and alginate dressings in terms of healing rate, shorter treatment time and the number of dressing changes [33].

However, due to the multiple aspects involved in the management of the PUs, such as moist environments, heat accumulation and the risk of infections, the design of an advanced wound dressing with specific and simultaneous features can be of great interest for PU prevention. This was the goal of this research, which aimed at providing a novel approach based on nanomaterials to improve the performance of conventional wound dressings through the combination of different nanotechnologies never tested together before and never explored for this specific application. Hence, for this specific application, multilayer A comprises St-St as an inner layer with high antibacterial capability and hydrophilic features, which are useful for the prevention of bacterial contamination and absorption of sweat/moisture in contact with the skin; meanwhile, a more hydrophobic external layer, as in SAt-St, can protect the skin from external factors. In the multilayer system B, the addition of an intermediate layer, FTBt, offers a good compromise between conductivity and antibacterial efficacy.

## 4. Conclusions

In order to prevent the development of pressure ulcers, three technologies have been applied for the treatment of cotton gauzes. Specifically, coatings based on nanometric silver, ashes and functionalized torrefied biomass, each deposited using a specific technology, have shown to be effective in terms of both antibacterial and thermal conductivity properties. In particular, the microbiological tests confirmed the antibacterial efficacy of the silver-based treatment and, as expected, the absence of antibacterial efficacy of the silane–ashes-based coating. Good results were obtained for the FTBt samples. The additional silver treatment improved the antibacterial efficacy in all the samples. Hydrophobicity was demonstrated only by SAt samples, with contact angle values higher than 110°, which decreased after the second silver treatment. SAt samples demonstrated the highest thermal conductivity, further improved by the second silver-based treatment, which was considered necessary in this project for the development of two models of multilayer systems. Multilayer A has been designed, from inside to outside, by using St-St and SAt-St fabric layers, while Multilayer B has been designed, from inside to outside, by St-St, FTBt-St and SAt-St fabric layers. The systems are intended to provide high antibacterial and hydrophilic properties in contact with skin and ensuring protective properties due to hydrophobic and conductive behaviour on the outer side. Moreover, the addition of an intermediate layer in the B-system represents a good compromise in terms of conductivity and antimicrobial effectiveness.

## Figures and Tables

**Table 1:** Thermal conductivity values of untreated and treated fabrics.

Sample	Thermal Conductivity (W/mK)
untreated gauze	0.15 ± 1.8 × 10^−3^
gauze_SAt	0.36 ± 3.9 × 10^−3^
gauze_FTBt1	0.15 ± 1.3 × 10^−3^
gauze_FTBt2	0.17 ± 1.8 × 10^−3^
gauze_St	0.19 ± 2.3 × 10^−3^
gauze_St-St	0.26 ± 3.8 × 10^−3^
gauze_SAt-St	0.38 ± 2.5 × 10^−3^
gauze_FTBt1-St	0.27 ± 3.2 × 10^−3^
gauze_FTBt2-St	0.28 ± 3.7 × 10^−3^

**Table 2:** Total thermal conductivity calculated for the multilayer systems.

Multilayer System	Thickness (m)	Total Thermal Resistance (k/W)	Total Thermal Conductivity (W/mK)
A	8.4 × 10^−4^	2.7 × 10^−3^	3.1 × 10^−1^
B	1.0 × 10^−3^	3.4 × 10^−3^	2.9 × 10^−1^

## Data Availability

The literature data supporting the discussion are reported as references in the reference list.
